# Guselkumab-induced vitiligo in a patient with psoriatic arthritis

**DOI:** 10.1016/j.jdcr.2023.08.043

**Published:** 2023-09-14

**Authors:** Fabrizio Martora, Maria Carmela Annunziata, Luigi Guerriero, Teresa Battista, Luca Potestio, Matteo Megna

**Affiliations:** Dermatology Unit, Department of Clinical Medicine and Surgery, University of Naples Federico II, Naples, Italy

**Keywords:** guselkumab, psoriatic arthritis, vitiligo

## Introduction

In recent years, the use of interleukin (IL)-23 inhibitors in the treatment of psoriatic arthritis (PsA) has been the subject of extensive research. By specifically binding to the p19 subunit of IL-23, IL-23 inhibitors block downstream signaling pathways inhibiting inflammatory responses. To date, guselkumab—a fully human monoclonal antibody targeting IL-23p19 subunit—has been approved for adult moderate-to-severe plaque psoriasis and PsA.^1^^,^^2^ Vitiligo is a skin disorder clinically characterized by depigmented macules and patches, most frequently localized in periocular area, hands, knees, and genitals. The disease is caused by selective autoimmune destruction of melanocytes.^3^

## Case report

**Fig 1:**
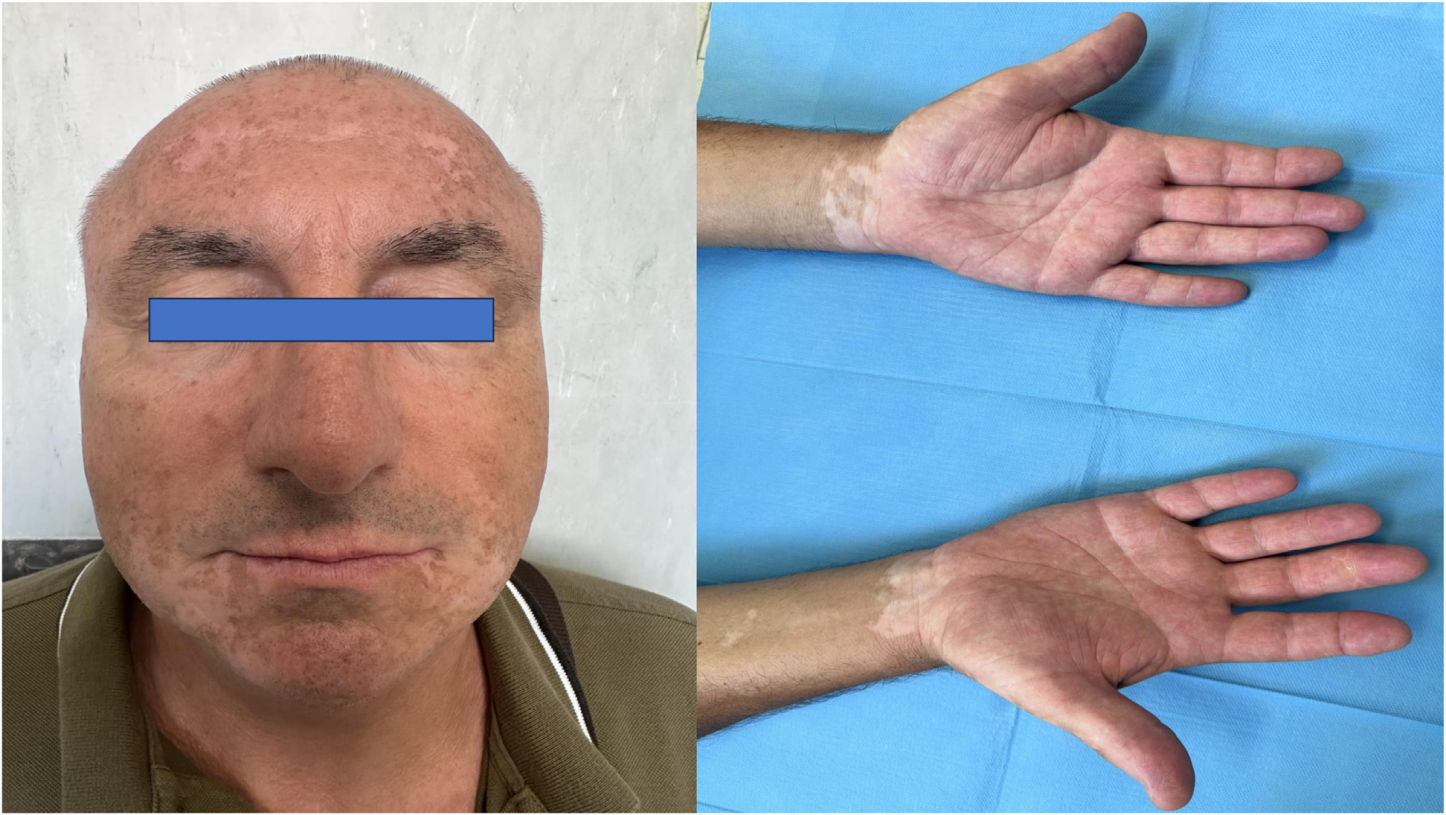
Hypopigmented macules and patches (maximum dimension, 3 × 4 cm) appeared on the facial area, particularly on cheeks and chin, saving periocular area during the treatment with guselkumab.

Herein, we present the case of a 60-year-old patient with 10-year history of severe PsA. The patient had been treated with methotrexate and sulfasalazine for several months. These drugs had been discontinued because of transaminitis. Subsequently, he started receiving etanercept for 12 weeks without improvement. He then received golimumab from May 2019 to October 2021 and secukinumab for 2 years and discontinued both because of loss of efficacy. Therefore, he started therapy with guselkumab in March 2023 with good clinical results. After 3 months, a Disease Activity in Psoriatic Arthritis score of 4 was reached, indicating disease remission. Although clinical response remained stable over time, 6 weeks later, hypopigmented macules and patches (maximum dimension, 3 × 4 cm) appeared on the facial area, particularly on cheeks and chin, sparing periocular area (Fig 1). Wood lamp examination revealed fluorescent achromatic patches, confirming the diagnosis of vitiligo. The patient does not have a positive family history of vitiligo, and no personal or familiar concomitant autoimmune diseases were registered. Blood tests for thyroid disease, diabetes, celiac disease, or anemia were negative. Treatment with topical calcineurin inhibitors was started without guselkumab suspension. No data regarding follow-up visits are available.

## Discussion

Vitiligo has been reported previously as an idiosyncratic adverse effect of anti–tumor necrosis factor-α agents, including adalimumab. Although the mechanism of correlation is not known, it is hypothesized that anti–tumor necrosis factor-α is a cytokine involved in the pathogenesis of vitiligo.^4^ More recently, the advent of drugs inhibiting the Th17 pathway has brought new reports of paradoxical adverse events. A few reports of vitiligo onset during anti-IL-17 treatment (ixekizumab or secukinumab) were reported, and in none of these cases, the biologic drug was interrupted.^5^, ^6^, ^7^, ^8^

Recently, Lee et al^8^ reviewed the literature regarding the use of Janus kinase inhibitors and anti-IL-23 in the treatment of vitiligo.

Regarding the use of anti-IL-23, several studies have reported an increase of IL-23 levels in patients with vitiligo; therefore, the authors concluded that considering the established documentation of IL-23 elevation in patients with vitiligo, targeted treatment should be considered as a potential novel therapy.^8^

IL-23 is an important cytokine in autoimmunity, promoting Th17 cell differentiation and causing an inflammatory response, leading to the production of neutrophils; therefore, the authors concluded that the role of these drugs in the treatment of vitiligo could be considered.^8^

There have been no reported cases of the occurrence of vitiligo during anti-IL-23 therapy so far; to the best of our knowledge, our case is the first to described in the literature. It could be hypothesized as a pathogenetic mechanism that IL-23 inhibition may cause Th1/Th17 polarization by increasing the expression of IL-17 and other cytokines involved in the pathogenesis of vitiligo.^9^^,^^10^ New studies in a wide range of patients will be needed in the future to establish this pathogenetic hypothesis.

## Conflicts of interest

None disclosed.
